# Work-related support for employed and self-employed people with rheumatoid arthritis or axial spondyloarthritis: a cross-sectional online survey of patients

**DOI:** 10.1007/s00296-024-05643-z

**Published:** 2024-06-20

**Authors:** N. F. Bakker, S. F. E. van Weely, A. Boonen, T. P. M. Vliet Vlieland, J. Knoop

**Affiliations:** 1 Department of Orthopedics, Rehabilitation and Physical Therapy, Leiden University Medical Center, Albinusdreef 2, P.O.Box 9600, 2300 RC Leiden, the Netherlands; 2 Institute of Allied Health Professions, HU University of Applied Sciences, Utrecht, the Netherlands; 3 Division of Rheumatology, Department of Internal Medicine, Maastricht University Medical Center, Maastricht, The Netherlands; 4 Care and Public Health Research Institute (CAPHRI), Maastricht University, Maastricht, The Netherlands; 5 Musculoskeletal Rehabilitation Research Group, HAN University of Applied Sciences, Nijmegen, the Netherlands

**Keywords:** Surveys and questionnaires, Work, Vocational rehabilitation, Rheumatoid arthritis, Axial spondyloarthritis, Cross-sectional study

## Abstract

**Background:**

Little is known about the provision of work-related support for (self-)employed people with rheumatoid arthritis (RA) or axial spondyloarthritis (axSpA) by healthcare providers (HCPs) or employers.

**Objective:**

This study aims to explore the experiences of (self-)employed people with RA or axSpA regarding work-related support from HCPs and employers in the Netherlands.

**Methods:**

This cross-sectional study concerned an online survey for (self-)employed people, aged ≥ 16 years and diagnosed with RA or axSpA. The survey focused on experiences with HCPs and employers’ work-related support and included questions on sociodemographic factors, health and work characteristics and work-related problems.

**Results:**

The survey was completed by 884 participants, 56% with RA and 44% with axSpA, of whom 65% were employed, 8% self-employed and 27% not employed. In total, 95% (589/617) of (self-)employed participants reported work-related problems. Sixty-five percent of employed and 56% of self-employed participants had discussed these work-related problems with rheumatologists and/or other HCPs. Whereas 69% of employees with their employer. Both employed and self-employed participants reported that work-related advices or actions were more often provided by other HCPs (53%) than rheumatologists (29%). Fifty-six percent of employees reported this work-related support by the employer.

**Conclusion:**

This survey among (self-)employed people with RA or axSpA found that the majority reported work-related problems, but only half of them received any work-related support for these problems. Discussion of work-related problems with HCPs was more often reported by employed than self-employed participants. More attention from especially rheumatologists and other HCPs is important to identify and address work-related problems promptly.

**Supplementary Information:**

The online version contains supplementary material available at 10.1007/s00296-024-05643-z.

## Introduction

Rheumatoid arthritis (RA) and axial spondyloarthritis (axSpA) are chronic inflammatory diseases, characterized by joint pain, stiffness, fatigue [1–4], limitations in daily activities, restrictions in societal participation and therefore reduced health-related quality of life [2, 5]. In the last decades, breakthroughs in pharmacological treatments have resulted in improved physical functioning and societal participation, including work, both in people with RA and axSpA [6, 7]. However, the literature has repeatedly shown that a considerable proportion of (self-)employed people with RA [8, 9] or axSpA [7, 9] still experience restrictions in work participation when compared to the general population. These restrictions include reduced productivity while at work (presenteeism) and sick leave or work disability (absenteeism). This reduced work participation of people with RA or axSpA was found to pose a substantial economic burden on both individuals and society [5, 10].

According to the 2021 EULAR Points to consider to support people with rheumatic and musculoskeletal diseases to participate in healthy and sustainable paid work [11], supporting people in maintaining employment is a shared responsibility of all stakeholders. These stakeholders may include the treating rheumatologist or specialized rheumatology nurse, other healthcare providers (HCP) but also the employer or the occupational physician. Work-related support may include advices or actions to reduce work-related problems and be provided by single professionals or a multidisciplinary team, either or not in a vocational rehabilitation program.

Regarding the extent to which work-related support is actually received in daily practice, the literature is scarce. In RA, a French survey among 81 employed patients or patients looking for a job reported that in about half of the consultations with the rheumatologists work-related problems were discussed [12]. In addition, a Dutch survey in 78 employed RA patients most frequently reported work-related support from the rheumatologist (36%), occupational physician (30%) and occupational therapist (27%) [13]. In axSpA, a German survey study among 695 patients who were employed or were not employed due to axSpA reported that 23% ever participated in vocational rehabilitation programs, and that this was initiated in 21% by the rheumatologist and 18% by the general practitioner [14].

The abovementioned studies varied considerably regarding their selection of patients (employment status and presence of a work-related problems) and their scope regarding the various sources of support (rheumatologist, multiple professionals or vocational rehabilitation programs). Thereby, comprehensive insight into the actual support employed people with RA or axSpA may receive from all potential professionals involved is lacking. Also, for those who are working, it is usually important to differentiate between employed and self-employed, as the available resources for support may differ between these groups. Getting more insight into the experiences of employed and self-employed people with RA or axSpA regarding work-related support from rheumatologists, other HCPs and employers if applicable, is important to identify bottlenecks and improve work-related support for these people if necessary. Therefore, this cross-sectional survey study aims to describe the experiences of Dutch (self-)employed people with RA or axSpA regarding their work-related problems and the work-related support they received from rheumatologists, other HCPs, or, if applicable, their employer.

## Methods

### Study design

A cross-sectional, nationwide survey study was conducted in people with RA or axSpA. Ethical approval to conduct the study was obtained from the Medical Ethics Committee Leiden The Hague Delft (W.23.007). This study was conducted in agreement with the declaration of Helsinki [15] and in compliance with the General Data Protection Regulations and the Dutch Medical Research Involving Human Subjects Act. All participants provided informed consent for their participation. As recommended by the Enhancing the QUAlity and Transparency Of health Research (EQUATOR) network, this study is reported in line with the Checklist for Reporting Results of Internet E-Surveys (CHERRIES) [16] and the STrengthening the Reporting of OBservational studies in Epidemiology (STROBE) checklist [17] (see Appendix 2 for the filled checklists).

### Participants

People were eligible for participation in the study if they were aged ≥ 16 years and reported a physician-confirmed diagnosis of RA or axSpA. Our rationale for this age limit is that education in the Netherlands is compulsory until the age of 16 [18]. After this age, people can enter the labour market. Furthermore, axSpA typically affects people from a young age [19]. People with a paid job (currently or in the past) could participate in the study. Participants were recruited via a link to the survey on the websites and social media channels of the Dutch Arthritis Society (ReumaNederland), the Dutch axial SpA foundation (Stichting axiale SpA Nederland), the Dutch foundation for adolescents with rheumatic diseases (Youth-R-Well), an online journal for people with rheumatic diseases (ReumaMagazine) and on nine Facebook groups for people with RA or axSpA. The survey was disseminated between May 19th and September 4th, 2023. Because of the descriptive character of this study, a sample size calculation was considered not applicable. We used a convenience sampling method and roughly aimed for a minimum of 800 respondents, as we considered this as necessary to reach sufficient numbers of participants within each category of work status and disease.

### Study context

In the Netherlands, rheumatologists and other HCPs, such as general practitioners and physiotherapists, share the responsibility of identifying work-related problems in people with RA or axSpA to facilitate timely work-related support [11, 20, 21]. In the Netherlands, occupational HCPs, including occupational physicians and labour experts, can provide work-related support for employees. According to the Dutch Eligibility for Permanent Incapacity Benefit (restrictions) Act, employers are obliged to offer their employees on prolonged sick leave (i.e., ≥ 6 weeks) a consultation with an occupational physician [22–24]. Moreover, employees can approach an occupational HCP themselves in case they want work-related support as a preventive measure in the event of work-related problems or short- term sick leave [22]. Self-employed people have no occupational physician and are only advised to have a sick leave and/or disability insurance [25].

Self-employed people with a sick leave and/or disability insurance are entitled to comparable work-related support as employed people. As a consequence of being uninsured, self-employed people may have no income during their sick leave or in case of job loss.

### Survey

The survey (see Appendix 1) consisted of 43 items, of which 10 items (i.e., items on education level, comorbidities, employment type, job sector, size of company and working hours) were based on existing, validated items from the Dutch Central Bureau of Statistics (CBS) [26, 27] and two items (i.e., items on work ability and health-related quality of life) were validated outcome measures [28, 29]. The other 31 items were generated by the authors based on previous qualitative studies regarding work-related support for people with RA or axSpA [12–14, 24, 30]. In the pre-testing phase, the survey was presented to the research team (four researchers with experience in qualitative and quantitative research) in order to provide input and recommendations on the wording, clarity, and comprehension of the items. Twenty laypersons from the general population then completed the survey to assess how items were interpreted by the target population (e.g. face validity [31]). Items that laypersons found unclear were reformulated or removed from the survey after consultation with the research group**.**

The survey focused on experiences of (self-)employed participants with work-related problems concerning work-related support from HCPs and employers. Furthermore, items on the participants’ sociodemographic and health characteristics, work characteristics and work-related problems were included. The sociodemographic and health characteristics were collected from all participants, while the work characteristics and work-related problems were only gathered from current (self-)employed participants. All (self-)employed participants with work-related problems filled in the items about experiences with the rheumatologist and other HCPs. Employed participants also completed items about their experiences with their employer. The survey was administered via an online link using the OnlinePROMs® software (2020; Interactive Studios, conform ISO27001 and NEN7510). The completion of the survey took approximately 15 min to finish, and the results could not be linked to an IP address.

#### Sociodemographic and health characteristics

Sociodemographic characteristics included age, sex, educational level, including low, medium and high [26], and whether participants (partly) stopped working due to the disease (yes/no). The health characteristics included self-reported diagnosis of RA or axSpA, self-reported disease duration and the number of comorbidities in the past 12 months, including diabetes, cardiovascular diseases, pulmonary diseases, cancer, migraine or severe headache, gastrointestinal complaints, musculoskeletal pain not related to RA/axSpA, allergy, urinary incontinence, liver diseases, kidney diseases, depression, obesity, Parkinson’s disease, and multiple sclerosis [26]. To assess the overall health-related quality of life (HRQoL) of participants, the validated Dutch translated EuroQol- 5 Dimensions 5-Level (EQ-5D-5L) was administered [28, 32]. The EQ-5D-5L comprises five items covering the dimensions mobility, self-care, activities of daily living, pain/discomfort and anxiety/depression. The Dutch health utility score ranges from − 0.446 to 1.000, with a score of 1 defining full health and a score below zero representing a health state worse than death [28, 32, 33]. In addition, participants completed the EQ-visual analogue scale (VAS) on their current health status, ranging from 0 (worst) to 100 (best).

#### Work characteristics and work-related problems

Items on work characteristics included employment type (employed/self-employed), job sector, including agricultural, craftmanship, transport, administrative, commercial, service and other size of company (categories of number of employees) and working hours (categories in hours/week), and were based on similar items used in national surveys [27]. Occupational class was determined according to the International Standard Classification of Occupations 2008 (ISCO-08) [34].The classification considers nine major occupational classes, which were further categorized, based on a previous study [35], into two categories: white-collar workers (i.e. managers, teachers, service and sales workers) and blue-collar workers (i.e. agricultural, forestry and fishery workers, cleaners).

Items on work-related problems included existence of work-related problems due to RA or axSpA in the past 12 months (yes/no) and if present, their type, including fatigue, pain in joints/muscles, stiffness of joints/muscles, morning or starting stiffness, difficulty moving and swollen joints, presence of current sick leave due to RA or axSpA (yes/no) and duration of current sick leave (categories in months). The self-reported work ability ofparticipants was assessed with the Work Ability Index-Single Item Scale (WAS) [29, 36]. The WAS is a responsive outcome measure to assess the status and progress of work ability and is highly predictive for future sick leave. It consists of a numeric rating scale (NRS) indicating the level of work ability an individual experiences at the moment of measuring ranging from 0 = completely unable to work at all, to 10 = work ability at its best, and distinguishing the following well-accepted categories: 0–5 = poor, 6–7 = moderate, 8–9 = good, and 10 = excellent work ability [29].

#### Experiences with work-related support from rheumatologists, other HCPs and employers

The items on experiences of participants in the past 12 months were distinguished by three groups: experiences with discussing work-related problems with (1) a rheumatologist or specialized rheumatology nurse, (2) other HCP(s),such as physiotherapists, general practitioners, occupational physicians, and (3) the employer (not applicable for self-employed participants). The items comprised for each group: discussed work-related problems in the past 12 months (yes/no), if not: reasons for not discussing work-related problems and if yes: advices or actions arisen from discussing problems. Additionally, in group 1 (rheumatologist or specialized rheumatology nurse), a question about whether the participant had a rheumatology consultation in the past 12 months was included. Furthermore, in group 2 (other HCPs), participants were asked if the advices or actions they received from the HCP were sufficient (yes/no).

### Analysis

Descriptive statistics were used to analyze the data of the total sample, and separately for the employed, self-employed and not employed participants and RA and axSpA populations. Normally distributed continuous variables were presented as means with standard deviations (SD) and variables with a skewed distribution as medians with interquartile ranges (IQR). Categorical variables were shown as frequencies with percentages. All statistical analyses were performed with IBM SPSS Statistics for Windows, version 29.0 (IBM Corp, Armonk, NY, USA).

## Results

**Fig. 1:**
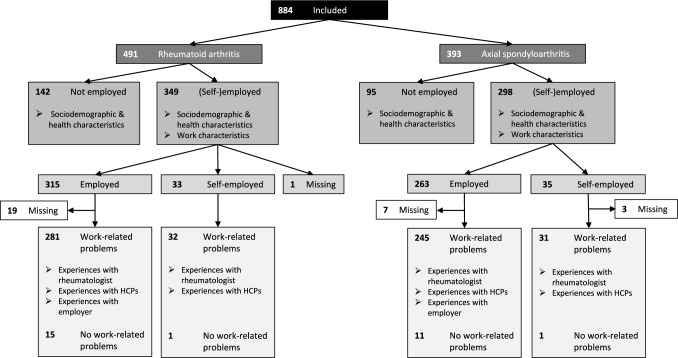
Flowchart of the participants in the survey

In total 1,339 people opened the survey, of whom 939 provided informed consent. Fifty-five people closed the survey after providing informed consent, resulting in 884 surveys available for analysis. Of the participants, 491/884 (56%) reported a diagnosis of RA and 393/884 (44%) of axSpA (Flowchart, Fig. 1).

### Sociodemographic and health characteristics

Table 1 shows the characteristics of the participants. About three-quarters of the participants with RA (n = 349/491; 71%) and axSpA (298/393; 76%) had a paid job, of whom less than 10% was self-employed (33/491; 7% in RA and 35/393; 9% in axSpA). Of the not employed participants, the majority (101/133; 76% in RA and 83/95; 87% in axSpA) had stopped working due to their disease. The mean (SD) EQ-5D-5L of (self-)employed participants was 0.69 (0.19) for RA and 0.60 (0.24) for axSpA respectively, whereas the EQ-5D-5L of not employed people was substantially lower: 0.48 (0.26) for RA and 0.38 (0.26) for axSpA. In both groups, the majority (247/303; 82% in RA and 240/271; 89% in axSpA) of participants had one or more comorbidities.

**Table 1:** Characteristics of employed and not employed participants with rheumatoid arthritis or axial spondyloarthritis participating in a cross-sectional survey study

	Rheumatoid arthritis, N = 491			Axial spondyloarthritis, N = 393		
	*Total,* *n* = *491*	*(Self-)employed, n* = *349*	*Not employed, n* = *142*	*Total,* *n* = *393*	*(Self-)employed, n* = *298*	*Not employed, n* = *95*
	n = 398	n = 285	n = 113	n = 317	n = 233	n = 84
**Age, years; mean (SD)**	49.4 (12.0)	48.2 (10.6)	52.4 (14.6)	46.3 (10.6)	45.3 (10.6)	49.3 (11.4)
Pensionable age; n (%)*	17 (4.3%)	0 (0.0%)	17 (15.0%)	6 (1.5%)	1 (0.4%)	5 (6.0%)
	n = 405	n = 289	n = 116	n = 324	n = 240	N = 84
**Sex, female; n (%)**	359 (88.6%)	263 (91.0%)	96 (82.8%)	269 (83.0%)	198 (82.5%)	71 (84.5%)
	N = 400	N = 287	N = 113	N = 324	N = 240	N = 84
**Self-reported disease duration¤, years;** **median (IQR)**	7.0 (3.0–17.0)	7.0 (3.0–16.0)	10.0 (4.0–19.0)	7.0 (3.0–14.0)	7.0 (3.0–13.0)	8.5 (4.0–19.0)
**Educational level#; n (%)** Low Medium High	N = 406 27 (6.7%) 174 (42.8%) 205 (50.5%)	N = 289 15 (5.2%) 108 (37.4%) 166 (57.4%)	N = 117 12 (10.3%) 66 (56.4%) 39 (33.3%)	N = 323 18 (5.6%) 144 (44.6%) 161 (49.8%)	N = 239 12 (5.0%) 97 (40.6%) 130 (54.4%)	N = 84 6 (7.1%) 47 (56.0%) 31 (36.9%)
**Comorbidities; n (%)** 0 1 2 3 or more	N = 303 56 (18.5%) 117 (38.6%) 71 (23.4%) 59 (19.5%)	N = 210 42 (20%) 92 (43.8%) 40 (19.0%) 36 (17.1%)	N = 93 14 (15.1%) 25 (26.9%) 31 (33.3%) 23 (24.8%)	N = 271 31 (11.4%) 104 (38.4%) 57 (21.0%) 79 (29.1%)	N = 189 25 (13.2%) 84 (44.4%) 38 (20.1%) 42 (22.2%)	N = 82 6 (7.3%) 20 (24.2%) 19 (23.2%) 37 (45.1%)
**Stopped working due to disease; n (%)** Yes			N = 133 101 (75.9%)			N = 95 83 (87.4%)
**EQ-5D-5L, range -0.5 to 1; mean (SD)**	n = 411 0.63 (0.23)	n = 290 0.69 (0.19)	n = 121 0.48 (0.26)	n = 329 0.54 (0.26)	n = 243 0.60 (0.24)	n = 86 0.38 (0.3)
**EQ-VAS, range 0 to 100; mean (SD)**	n = 411 62.5 (17.6)	n = 290 65.9 (16.1)	n = 121 54.2 (18.3)	n = 329 58.0 (17.2)	n = 243 61.0 (16.9)	N = 86 49.5 (15.3)

*EQ-5D-5L* EuroQol- 5 Dimensions 5-Level, *EQ-VAS* EuroQol-visual analogue scale

^*^Pensionable age is ≥ 67 years

^¤^Non-normal distributed

^#^Education level; low = primary school or lower vocational education; medium = lower general secondary school or intermediate vocational education; high = higher general secondary school, higher vocational education, or university

### Work characteristics and work-related problems

Table 2 presents the work characteristics and work-related problems for the total group of (self-) employed participants, because results were very similar between RA and axSpA (results for RA and axSpA separately are displayed in supplementary Tables A-D). Most (self-)employed participants (578/627; 92%) had white-collar jobs. The majority (589/617; 95%) experienced work-related problems in the past 12 months due to the RA or axSpA. The most frequently reported work-related problems were fatigue (563/589; 96%) and pain in muscles or joints (552/589; 94%). The mean (SD) score on the WAS was 6.4 (1.8) for (self-)employed participants, indicating moderate work ability. In total, (196/617) 32% of (self-)employed participants with RA or axSpA had a WAS score < 6, indicating poor work ability.

**Table 2:** Work characteristics and work-related problems of (self-)employed participants with rheumatoid arthritis or axial spondyloarthritis

	(Self-)employed participants with rheumatoid arthritis and axial spondyloarthritis		
	*Total,* *n* = *646*	*Employed, n* = *578*	*Self-employed, n* = *68*
**Employment type; n (%)** Permanent employment Temporary employment Self-employed with personnel Self-employed without personnel	N = 646 519 (80.3%) 59 (9.1%) 8 (1.2%) 60 (9.3%)	N = 578 519 (89.8%) 59 (10.2%) – –	N = 68 – – 8 (11.8%) 60 (88.2%)
**Top-3 job sector; n (%)** Healthcare Education and training Retail and sales	N = 646 241 (37.3%) 76 (11.8%) 64 (9.9%)	N = 578 219 (37.9%) 71 (12.3%) 59 (10.2%)	N = 33 22 (32.4%) 5 (7.4%) 5 (7.4%)
**Occupational class; n (%)** White-collar Blue-collar	N = 627 578 (92.2%) 49 (7.8%)	N = 561 516 (92.0%) 45 (8.0%)	N = 66 62 (93.9%) 4 (6.1%)
**Size of company; n (%)** < 10 employees 10 to 50 employees 51 to 250 employees > 250 employees	N = 646 107 (16.6%) 111 (17.2%) 106 (16.4%) 322 (49.8%)	N = 578 49 (8.5%) 107 (18.5%) 104 (18.0%) 318 (55.0%)	N = 68 58 (85.3%) 4 (5.9%) 2 (2.9%) 4 (5.9%)
**Working hours; n (%)** < 12 h/week 12 to 24 h/week 25 to 36 h/week > 36 h/week	N = 646 37 (5.7%) 244 (37.8%) 237 (36.7%) 128 (19.8%)	N = 578 31 (5.4%) 221 (38.2%) 217 (37.5%) 109 (18.9%)	N = 68 6 (8.8%) 23 (33.8%) 20 (29.4%) 19 (27.3%)
**Work-related problems in the past 12 months due to the disease; n (%)** Yes	N = 617 589 (95.5%)	N = 552 526 (95.3%)	N = 65 63 (96.9%)
**Type of work-related problems due to disease; n (%)** *(Multiple answers possible)* Fatigue Pain in joints/muscles Stiffness of joints/muscles Morning or starting stiffness Difficulty moving Swollen joints	N = 589 563 (95.6%) 552 (93.7%) 525 (89.1%) 493 (83.7%) 465 (78.9%) 245 (41.6%)	N = 526 503 (95.6%) 491 (93.3%) 470 (89.3%) 437 (83.1%) 415 (78.9%) 222 (42.2%)	N = 63 31 (49.2%) 32 (50.8%) 28 (44.4%) 26 (41.3%) 27 (42.9%) 17 (27.0%)
**Currently on sick leave due to disease; n (%)** Yes, on partial sick leave Yes, on full sick leave	N = 589 58 (9.8%) 43 (7.3%)	N = 526 54 (10.3%) 43 (8.2%)	N = 63 4 (6.3%) -
**Duration of current sick leave due to disease; n (%)** < 6 months 6 to 12 months > 12 months	N = 101 39 (38.6%) 32 (31.7%) 30 (29.7%)	N = 97 39 (40.2%) 29 (29.9%) 29 (29.9%)	N = 4 - 3 (75.0%) 1 (25.0%)
**Work Ability Index-Single Item Scale (WAS),** **range 0 to 10; Mean (SD)** **WAS categories; n (%)** Poor work ability (0–5) Moderate work ability (6–7) Good work ability (8–9) Excellent work ability (10)	N = 617 6.4 (1.8) 196 (31.8%) 298 (48.3%) 118 (19.1%) 5 (0.8%)	N = 552 6.5 (1.8) 174 (31.5%) 262 (47.5%) 112 (20.3%) 4 (0.7%)	N = 65 5.9 (1.6) 22 (33.8%) 36 (55.4%) 6 (9.2%) 1 (1.5%)

### Contacts with HCPs and employers

#### Rheumatologists and specialized rheumatology nurses

Table 3 shows the experiences of participants with discussing their work-related problems with the rheumatologist or specialized rheumatology nurse. In total, (309/534) 58% of (self-)employed participants who visited a rheumatologist or specialized rheumatology nurse in the past 12 months and experienced a work-related problem, discussed their problem with these HCPs. The most reported reason for not discussing work-related problems was ‘I did not think this was necessary’ for (143/225) 64% of the participants and ‘I had not thought of this’ for (114/225) 51%. Twenty-nine percent (90/309) of (self-)employed participants reported that discussing work-related problems had led to advices or actions to reduce these problems. The advice to discuss possible adjustments to the job with the employer was the most (47/90; 52%) reported received advice.

**Table 3:** Experiences with discussing work-related problems with the rheumatologist or specialized rheumatology nurse

	Participants with rheumatoid arthritis or axial spondyloarthritis and work-related problems, N = 589		
	*Total,* *n* = *589*	*Employed, n* = *526*	*Self-employed, n* = *63*
**Rheumatology consultation in the last 12 months; n (%)** Yes, with rheumatologist Yes, with specialized rheumatology nurse	N = 566 444 (78.4%) 90 (15.9%)	N = 504 394 (78.2%) 83 (21.8%)	N = 62 50 (80.6%) 7 (19.4%)
**Discussed work-related problems with the rheumatologist or specialized rheumatology nurse; n (%)** Yes	N = 534 309 (57.9%)	N = 477 278 (58.3%)	N = 57 31 (54.4%)
**Reasons for not discussing work-related problems with the rheumatologist or specialized rheumatology nurse; n (%)** *(Multiple answers possible)* I did not think this was necessary I had not thought of this I did not think they could help me with this I found it difficult to discuss this I did not had time for this I was afraid that my employer would be informed	N = 225 143 (63.5%) 114 (50.7%) 84 (37.3%) 53 (23.6%) 51 (22.7%) 13 (5.8%)	N = 199 128 (64.3%) 100 (50.2%) 71 (35.7%) 47 (23.6%) 42 (21.1%) 12 (6.0%)	N = 26 15 (57.7%) 14 (53.8%) 13 (50.0%) 6 (23.1%) 9 (34.6%) 1 (3.8%)
**Advices or actions arisen from discussing work-related problems with the rheumatologist or specialized rheumatology nurse; n (%)** Yes	N = 309 90 (29.1%)	N = 278 82 (29.5%)	N = 31 8 (25.8%)
**Advices or actions resulting from discussions with the rheumatologist or specialized rheumatology nurse; n (%)** *(Multiple answers possible)* I have been advised to discuss adjustments in working tasks/hours/environment with my employer I have been referred to a different HCP I received advice on how to perform my job with fewer problems I have been referred to an occupational HCP A workplace examination has been carried out	N = 90 47 (52.2%) 45 (50.0%) 39 (43.3%) 29 (32.2%) 12 (13.3%)	N = 82 46 (56.1%) 43 (52.4%) 36 (43.9%) 28 (34.1%) 11 (13.4%)	N = 8 1 (12.5%) 2 (25.0%) 3 (50.0%) 1 (12.5%) 1 (12.5%)

*HCP: healthcare professional*

#### Other HCPs

In Table 4 the experiences of participants with discussing work-related problems in the past 12 months with other HCPs than rheumatologists/specialized rheumatology nurses are presented. Work-related problems were discussed with other HCPs by employed (204/458; 44%) and self-employed (15/56; 27%) participants. The most frequently consulted other HCP was the physiotherapist in both employed (141/204; 69%) and self-employed (13/15; 87%) participants. The most reported reason (177/295; 60%) of (self-employed) participants for not discussing work-related problems was ‘I did not think this was necessary’. Discussing work-related problems with an HCP led to advices or actions to reduce these problems in employed (109/204; 53%) and self-employed (8/15; 53%) participants. The most reported advice (71/109; 65%) received by employed participants was to discuss adjustments to the job with the employer. Self-employed participants were most frequently (7/8; 87%) advised on how to perform the job with fewer problems. Discussing work-related problems with an HCP was sufficient to reduce work-related problems according (144/219) 66% of (self-)employed participants.

**Table 4:** Experiences with discussing work-related problems with other HCPs

	Participants with rheumatoid arthritis or axial spondyloarthritis and work-related problems, N = 589		
	*Total,* *n* = *589*	*Employed, n* = *526*	*Self-employed, n* = *63*
**Discussed work-related problems with another (occupational) HCP; n (%)** Yes	N = 514 219 (42.6%)	N = 458 204 (44.5%)	N = 56 15 (26.8%)
**Which (occupational) HCP is consulted for work-related problems; n (%)** *(Multiple answers possible)* HCPs: Physiotherapist General practitioner Occupational therapist Psychologist Social worker Physiatrist Occupational HCPs: Occupational physician Labour expert Specialized occupational physiotherapist Insurance physician	N = 219 154 (70.3%) 90 (41.1%) 33 (15.1%) 55 (25.1%) 27 (12.3%) 17 (7.8%) 114 (52.0%) 37 (16.9%) 16 (7.3%) 9 (4.1%)	N = 204 141 (69.1%) 88 (43.1%) 32 (15.7%) 51 (25.0%) 26 (12.7%) 17 (8.3%) 114 (55.9%) 36 (17.6%) 16 (7.8%) 8 (3.9%)	N = 15 13 (86.7%) 2 (13.3%) 1 (6.7%) 4 (26.7%) 1 (6.7%) - - 1 (6.7%) - 1 (6.7%)
**Reasons for not discussing work-related problems with (occupational) HCP; n (%)** *(Multiple answers possible)* I did not think this was necessary I had not thought of this I found it difficult to discuss this I did not think anybody could help me with this I did not know who to consult I did not had time for this I was afraid that my employer would be informed I did not had money for this	N = 295 177 (60.0%) 110 (37.3%) 107 (36.3%) 105 (35.6%) 95 (32.2%) 56 (19.0%) 46 (15.6%) 39 (13.2%)	N = 254 154 (60.6%) 93 (36.6%) 99 (39.1%) 88 (34.6%) 81 (31.9%) 46 (18.1%) 45 (17.7%) 34 (13.4%)	N = 41 23 (56.1%) 17 (41.5%) 8 (19.5%) 17 (41.5%) 14 (34.1%) 10 (24.4%) 1 (2.4%) 5 (12.2%)
**Advices or actions arisen from discussing work-related problems with the (occupational) HCP; n (%)** Yes	N = 219 117 (53.4%)	N = 204 109 (53.4%)	N = 15 8 (53.3%)
**Advices or actions resulting from discussions with the HCP; n (%)** *(Multiple answers possible)* I have been advised to discuss adjustments in working tasks/hours/environment with my employer I received advice on how to perform my job with fewer problems I have been referred to a (different) HCP I received a physical exercise program to be able to perform my job with fewer problems I have been referred to an (different) occupational HCP I received mental training to be able to perform my job with fewer problems A workplace examination has been carried out	N = 117 72 (61.5%) 74 (63.2%) 46 (39.3%) 41 (35.0%) 31 (26.5%) 27 (23.1%) 18 (15.4%)	N = 109 71 (65.1%) 67 (61.5%) 45 (41.3%) 34 (31.2%) 31 (28.4%) 26 (23.9%) 18 (16.5%)	N = 8 1 (12.5%) 7 (87.5%) 1 (12.5%) 7 (87.5%) - 1 (12.5%) -
**Was discussing the work-related problems with the (occupational) HCP sufficient to reduce the work-related problems; n (%)** Yes	N = 219 144 (65.8%)	N = 204 135 (66.2%)	N = 15 9 (60.0%)

*HCP: healthcare professional*

#### Employers

Table 5 shows the experiences of employed participants with discussing work-related problems with their employer in the past 12 months. Work-related problems were discussed with the employer by (352/514) 68% of the employed participants. The most reported reason for not discussing work-related problems of (108/162) 67% employed participants was ‘I did not think this was necessary’. Discussing work-related problems with the employer led to advices or actions to reduce these problems in (198/352) 56% of participants. The advice to consult an occupational HCP (100/198; 50%) was reported most frequently.

**Table 5:** Experiences of employed participants with work-related problems with discussing work-related problems with the employer*

	Employees with rheumatoid arthritis or axial spondyloarthritis and work-related problems, n = 526
**Discussed work-related problems with the employer; n (%)** Yes	N = 514 352 (68.5%)
**Reasons for not discussing work-related problems with the employer; n (%)** *(Multiple answers possible)* I didn’t think this was necessary I found it difficult to discuss this I was afraid of possible adverse consequences for my job or employment contract I thought my employer couldn’t help me with this I hadn’t thought of this	N = 162 108 (66.7%) 91 (56.2%) 87 (53.7%) 66 (40.7%) 34 (21.0%)
**Advices or actions arisen from discussing work-related problems with the employer; n (%)** Yes	N = 352 198 (56.2%)
**Advices or actions resulting from discussions with the employer; n (%)** *(Multiple answers possible)* I received advice to consult an occupational HCP I received advice to consult a HCP I perform fewer tasks in a working day I perform different tasks I work less hours: by reducing the number of hours in my employment contract I work less hours: by reducing taking vacation hours I work less hours: other I work at different times My work environment is adjusted I have found another job or am currently looking for another job	N = 198 100 (50.5%) 93 (47.0%) 82 (41.4%) 70 (35.3%) 32 (16.2%) 10 (5.0%) 39 (19.7%) 68 (34.3%) 78 (39.4%) 21 (10.6%)

^*^Not applicable for self-employed participants HCP: healthcare professionall

#### Overlap in contacts

**Fig. 2:**
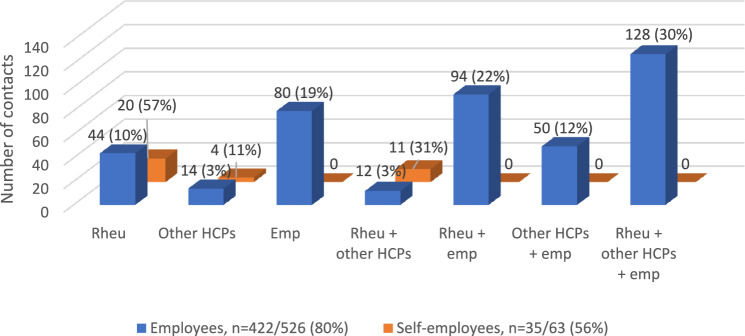
Overlap in contacts within rheumatologists (rheu), other HCPs and employers (emp) for discussing work-related problems with (self-)employed participants with RA or axSpA

In Fig. 2 the overlap in contacts between the three groups (rheumatologists, other HCPs and employers) for discussing work-related problems with (self-)employed participants with RA or axSpA is displayed. Work-related problems were discussed with at least one professional from the three groups by (422/526) 80% of employees. Self-employed participants discussed their work-related problems to a lesser extent, in (35/63) 56% with at least a rheumatologist or one other HCP.

## Discussion

This survey among Dutch (self-)employed people with RA or axSpA revealed that the majority (95%) encountered work-related problems, but only half of them reported to receive work-related support. Discussion of these reported work-related problems with HCPs was more often reported by employed than self-employed participants. Work-related support was most often initiated by HCPs other than the rheumatologist or specialized rheumatology nurse (for both employed or self-employed participants) or by the employer (for employed participants only).

The prevalence of work-related problems in our study (95%) is higher than in a previous study by Brown et al., which reported rates of 81% in (self-)employed people with RA and 73% in axSpA [37]. Furthermore, Garrido-Cumbrera et al., found that 68% of employed people with axSpA reported at least one work-related problem [38]. A cross-sectional study examining the impact of different diseases on work-related outcomes [39] found that in the context of multimorbidity, musculoskeletal diseases, including RA and axSpA, tended to increase the risk of adverse work outcomes and especially sick leave. Therefore, it is possible that people with RA or axSpA experience more work-related problems than other population with chronic illness. However, comparisons are hampered by differences in quantifying work-related problems. While our study assessed the presence of work-related problems by simply asking participants whether they experienced work-related problems, Brown et al. [37] assessed this with the Workplace Activity and Limitations Scale (WALS) [40]. Garrido-Cumbrera et al. [38] asked a similar question to us, but used different answer options. Possibly, the 10–25% higher work-related problem rates in our study could be explained by the lack of a question or answer option regarding fatigue in Brown et al. [37] and Garrido-Cumbrera et al. [38], as fatigue is the most commonly reported work-related problem in our study and was associated with presenteeism and job loss [41]. Furthermore, in a qualitative study, people with inflammatory arthritis identified fatigue as the most limiting factor on their work ability [42]. Research into the prevalence of fatigue as a work-related problem in working people with RA or axSpA is scarce. In inflammatory arthritis, several studies have shown that fatigue is frequently reported by patients, ranging from 40 to 80% [43], but this was not specified for the working population. In a working population of psoriatic arthritis patients, 51% were classified as having fatigue [44]. In the general workforce, approximately 20% of workers reported fatigue, while 40% of chronically ill workers reported fatigue [45]. Based on these studies, fatigue may be highly prevalent among the working population of people with inflammatory arthritis, but more research is needed to evaluate this. Moreover, it is difficult to compare our study with other studies because of differences in countries and years that the studies were conducted, selection of the study population, the recall period and the measures used to describe work-related problems. The majority of studies described the work participation burden of workers with RA or axSpA by measuring rates of job loss, absenteeism or presenteeism [8, 46, 47] instead of asking patients if they experienced work-related problems.

Our results indicate that more employed than self-employed people discuss their work-related problems with HCPs. Previous studies evaluated the frequency of discussing work-related problems with rheumatologists [12] or the provision of work-related support by HCPs to people with RA or axSpA [13, 14]. Furthermore, a scoping review from 2023, evaluating studies that aimed at medical specialties, including rheumatology, treating patients of working age, found that most medical specialists did not routinely discuss work with their patients [48]. However, none of these studies distinguished between employment types, therefore we cannot compare results. Non-necessity was the most frequently reported reason by self-employed participants for not discussing work-related problems with their rheumatologist or other HCP. It is possible that self-employed people might need less work-related support because they are more in control of organizing their job (e.g. working hours, tasks). It is also possible that self-employed people do not know what kind of work-related support is available from which HCP, as indicated by around half of the self-employed participants. Therefore, more research into the needs of self-employed people is necessary.

Appropriate guidance and work-related support from HCPs is recommended in clinical recommendations and guidelines [11, 20], but only about a half of the people who reported that they had discussed their work-related problems mentioned the receipt of work-related support. Regarding the initiation of work-related support, in employed participants, the employer was most often mentioned. Rheumatologists and specialized rheumatology nurses were least frequently reported to initiate work-related support by both employed and self-employed participants. This is in line with other studies, were support rates of 21–36% by rheumatologists for people with RA or axSpA were mentioned [13, 14]. Fortunately, about two-thirds of the (self-)employed participants reported that the work-related support they received from other HCPs had been sufficient to reduce work-related problems.

This survey study contributes to literature by describing the experiences of Dutch people with RA or axSpA regarding work-related support they received from their rheumatologist, other HCPs or employers. Moreover, our study is the first that described these experiences separately for employed and self-employed people, as their differing work setting may influence their needs for work-related support. Strengths of this study are the large sample sizes of 491 people with RA and 393 with axSpA, and the exploration of discussing work-related problems and subsequent provided work-related support from HCPs or employers. Our study included people with RA or axSpA, potentially limiting comparison to other cross-sectional studies. Moreover, our sample was relatively young, primarily female, highly educated and with a relatively low health-related quality of life. This might be a consequence of our study design characterized by self-selection through an online survey, requiring computer-literate participants, and the distribution via patient associations. Therefore, the results might not reflect the entire population of workers with RA or axSpA, limit generalizability and should be interpreted with caution. Further, the diagnosis was not verified by the rheumatologists, therefore some participants might not have an RA or axSpA diagnosis. However, compared to a survey study [37] that ensured a physician-confirmed diagnosis by selecting participants through rheumatology departments, our study group of (self-)employed RA and axSpA participants showed comparable characteristics like age, disease duration and employment status. Additionally, the participants were only asked whether the work-related support they received from other HCPs was sufficient but not whether they were satisfied with the support of the rheumatologist or employer. Hence, no statements could be made on the satisfaction of workers with work-related support from rheumatologists and employers. Fourthly, parts of the survey were developed by the research team and consisted of non-validated items and due to its quantitative design, participants were confined to pre-defined response options when answering the survey items. Nevertheless, the survey was pre-tested to enhance face validity and was improved accordingly. Finally, as in all surveys, participants could prematurely end the survey, resulting in different sample sizes per item. Possibly, these drop-outs distorted the overall picture.

Implications for future research include evaluating the prevalence of work-related problems and the frequencies of work-related discussions and support of HCPs and employers in a less selective population of RA and axSpA patients. For example, by including patients visiting the rheumatologist instead of via websites or social media channels to prevent self-selection. Furthermore, more research into different employment types, like employed and self-employed people, is needed to gain insight into the differences in discussing work-related problems and subsequent support. Moreover, this study was conducted in the Netherlands and due to differences in healthcare and social security systems between countries, comparable research should also be conducted in other countries to be able to make general recommendations to improve work-related support.

We would like to make some suggestions for improving work-related support for people with RA or axSpA, which focuses specifically on the Dutch system. All HCPs and especially rheumatologists and specialized rheumatology nurses should enhance their attention for work participation from the start of the diagnosis, to improve early identification of (self)-employed people with work-related problems. To achieve this, it might be essential to further educate HCPs about work-related problems and social security systems to facilitate discussions with (self-)employed patients and provide appropriate work-related support. Furthermore, people with RA or axSpA should become more aware that they can discuss work-related problems with their rheumatologist and other HCPs. Additionally, clinical guidelines should more explicitly highlight work disability issues and include recommendations for interventions aimed at improving work ability [11]. To facilitate discussions about work-related problems in clinical practice, specific tools (e.g., checklists, alerts) might be beneficial in reminding HCPs to address work-related issues.

## Conclusion

In this nationwide survey study, the majority of (self-)employed participants with RA or axSpA experienced work-related problems, but only half of them reported to receive work-related support. Discussion of work-related problems with HCPs was more often reported by employed than self-employed participants. Work-related support was more often initiated by employers than by HCPs. It seems important that HCPs and especially rheumatologists or specialized rheumatology nurses pay more attention to the presence of work-related problems and initiate discussions about work to enable timely work-related support and reduce restrictions in work participation and its societal impact.

### Supplementary Information

Below is the link to the electronic supplementary material.

Supplementary file1 (DOCX 32 KB)

Supplementary file2 (DOCX 27 KB)

Supplementary file3 (DOCX 43 KB)

## Declarations

### Open Data Sharing Note

The data generated during this study will not be publicly available, but will be available upon reasonable request to the corresponding author.

### Related Congress Abstract Publication

This study was selected by the European Alliance of Associations for Rheumatology (EULAR) Congress 2024 (12–15 June) for an oral abstract presentation. Online publication of the congress abstract will be provided by EULAR approximately two weeks prior to the congress.
