# Supplementary material for: Work-related support for employed and self-employed people with rheumatoid arthritis or axial spondyloarthritis: a cross-sectional online survey of patients
Source: Rheumatol Int. 2024 Jun 20;44(8):1553–65. doi: 10.1007/s00296-024-05643-z (PMC11222234; doi:10.1007/s00296-024-05643-z)
Supplement: Supplementary file 1 — Supplementary file1 (DOCX 32 KB) [file 296_2024_5643_MOESM1_ESM.docx]

**SCREENING**

I am 16 years or older.

□ Yes > next question

□ No > Thank you for completing. For this study, we are looking for individuals aged 16 years or older. > stop questionnaire

What rheumatic diagnosis has been established for you by a rheumatologist?

□ Rheumatoid Arthritis (RA)

□ Axial Spondyloarthritis (axSpA; including Ankylosing Spondylitis)

□ Other > Thank you for completing. For this study, we are looking for individuals diagnosed with RA or axSpA. > stop questionnaire

**INFORMED CONSENT**

I consent to the collection and use of my data for answering the research question in this study.

□ Yes > next question

□ No > Thank you for completing. For this study, you must consent to share your data. If you do not wish to, you cannot participate in the study. > stop questionnaire.

We kindly ask you to answer the questions as honestly as possible. Please read the question and answer options carefully. If you are unsure about an answer, it's okay. Just provide the answer that you think is correct for your situation. Most questions refer to the past 12 months. For some questions, you can provide further explanation if you wish.

1. **WORK SITUATION**

In this questionnaire, we use the abbreviation RA for Rheumatoid Arthritis and the abbreviation axSpA for Axial Spondyloarthritis (which also includes Ankylosing Spondylitis).

1. Do you currently have a paid job?

□ Yes > to question 5

□ No

2. Have you ever had a paid job in the past?

□ Yes

□ No > to question 38

3. Did you stop working because of your RA/axSpA?

□ Yes

□ No

4. Would you like to have a paid job again in the future?

□ Yes

□ No

5. How are you currently employed? (If you have multiple employment types, select the option in which you work the most hours)

□ As an employee with a permanent contract

□ As an employee with a temporary contract

□ As a self-employed entrepreneur with employees > then questions 18-21 do not apply

□ As a self-employed entrepreneur without employees > then questions 18-21 do not apply

6. In which sector do you work? (If you work in multiple sectors, select the option in which you work the most hours)

□ Agriculture, forestry, fishing
□ Mining, energy, water, waste

□ Construction

□ Manufacturing, installation, repair

□ Transportation, logistics, delivery services

□ Trade (e.g., retail, wholesale, car trade)

□ Hospitality, recreation, sports

□ Healthcare

□ Education

□ Government

□ IT, telecommunications, media

□ Financial services, real estate

□ Other, namely [FILL IN]

□ I don't know

7.What is your occupation or position? (Please be as specific as possible, e.g., by specifying a specialization or level. For example, not: mechanic. But: car mechanic) [FILL IN]

8. How many hours do you actually work per week? (Consider your average work situation in the past month, regardless of the number of hours per week in your contract/employment)

□ Less than 12 hours per week

□ 12 to 24 hours per week

□ 25 to 36 hours per week

□ more than 36 per week

9. How large is the company where you currently work?

□ Fewer than 10 employees ('micro enterprise')

□ Between 10 and 50 employees ('small enterprise')

□ Between 51 and 250 employees ('medium-sized enterprise')

□ More than 250 employees ('large enterprise')

**B. PROBLEMS WITH WORK PERFORMANCE**

10. If you were to rate your work ability at its best point in your life as a 10, what rating would you give your work ability AT THIS MOMENT? Check which rating applies:

0 1 2 3 4 5 6 7 8 10

0 = completely unable to work 10 = work ability at the best point in your life

11. Have you experienced problems in performing your work in the past 12 months due to your RA/axSpA and the symptoms caused by this condition?

□ Yes

□ No > to question 38

12. Are you currently on sick leave due to your RA/axSpA?

□ Yes

□ No > to question 15

13. How many percent (%) of the time are you on sick leave at your current job at this moment? ______ %

14. How long have you currently been (partially or fully) on sick leave from work due to your RA/axSpA?

□ 0-6 months

□ 6-12 months

□ Longer than 12 months

15.What problems have you experienced at work in the past 12 months due to your RA/axSpA? (select ‘yes’ if applicable to you and ‘no’ if not applicable to you)

□ Pain in joints/muscles ⃝ yes ⃝ no

□ Stiffness in joints/muscles ⃝ yes ⃝ no

□ Red or swollen joints ⃝ yes ⃝ no

□ Fatigue ⃝ yes ⃝ no

□ Difficulty with movement ⃝ yes ⃝ no

□ Morning or starting stiffness ⃝ yes ⃝ no

□ Other, namely [FILL IN]

16.Have you made adjustments to your work (such as changes in tasks or working hours) in the past 12 months due to problems performing your job?

□ Yes

□ No → go to question 18

17.What adjustments have you made to your work in the past 12 months? (select ‘yes’ if applicable to you and ‘no’ if not applicable to you)

▪ I perform fewer tasks in a workday ⃝ yes ⃝ no

▪ I perform different tasks ⃝ yes ⃝ no

▪ I work at different times ⃝ yes ⃝ no

▪ I work fewer hours ⃝ yes ⃝ no

▪ due to a reduction in the number of hours in my contract/employment ⃝ yes ⃝ no

▪ by taking vacation hours ⃝ yes ⃝ no

▪ other, namely: … ⃝ yes ⃝ no

▪ My work environment (e.g., desk, chair, mouse) has been adjusted ⃝ yes ⃝ no

▪ I have found another job or am currently looking for another job ⃝ yes ⃝ no

▪ Other, namely: …

1. **DISCUSSING WORK-RELATED ISSUES WITH EMPLOYER/SUPERVISOR**

[not applicable for self-employed people]

18.In the past 12 months, have you discussed the problems at work due to your RA/axSpA with your employer/supervisor?

□ Yes → go to question 20

□ No

19.What were your reasons for NOT discussing the problems at work due to your RA/axSpA with your employer/supervisor in the past 12 months? (select ‘yes’ if applicable to you and ‘no’ if not applicable to you)

▪ I did not find it necessary ⃝ yes ⃝ no

▪ I did not think of it ⃝ yes ⃝ no

▪ I thought my employer could not help me with this ⃝ yes ⃝ no

▪ I was afraid of possible negative consequences for my job or employment contract ⃝ yes ⃝ no

▪ I found it difficult to discuss ⃝ yes ⃝ no

▪ Other reason, namely: [FILL IN] → go to question 22

20.Has discussing the problems at work with your employer/supervisor led to actions (such as adjustments to your work, advice to visit a healthcare or labor professional) in the past 12 months?

□ Yes

□ No → go to question 22

21.What actions have resulted from discussing the problems at work with your employer/supervisor? (select ‘yes’ if applicable to you and ‘no’ if not applicable to you)

▪ I perform fewer tasks in a workday ⃝ yes ⃝ no

▪ I perform different tasks ⃝ yes ⃝ no

▪ I work at different times ⃝ yes ⃝ no

▪ I work fewer hours ⃝ yes ⃝ no

▪ due to a reduction in the number of hours in my contract/employment ⃝ yes ⃝ no

▪ by taking vacation hours ⃝ yes ⃝ no

▪ other, namely: … ⃝ yes ⃝ no

▪ My work environment (desk, chair, mouse, etc.) has been adjusted ⃝ yes ⃝ no

▪ I have found another job or am currently looking for another job ⃝ yes ⃝ no

▪ Advice to visit a healthcare professional (such as rheumatologist, physiotherapist) ⃝ yes ⃝ no

▪ Advice to visit a labor professional (such as occupational physician, labour expert) ⃝ yes ⃝ no

1. **DISCUSSING WORK-RELATED ISSUES DURING RHEUMATOLOGY APPOINTMENTS**

Due to your rheumatic condition, you probably have an appointment (in person, by phone, or online) with your rheumatologist or rheumatology nurse/nurse specialist at least once a year.

22.Have you had a rheumatology appointment in the past 12 months?

□ Yes

□ No → go to question 28

23.Who did you have your most recent rheumatology appointment with in the past 12 months?

□ Rheumatologist

□ Rheumatology nurse/nurse specialist

□ Other, namely [FILL IN]

Answer the following questions about this most recent rheumatology appointment.

24.Did you discuss the problems in performing your work due to your RA/axSpA during this rheumatology appointment?

□ Yes → go to question 26

□ No

25.What were your reasons for NOT discussing the problems at work due to your RA/axSpA during this rheumatology appointment? (select ‘yes’ if applicable to you and ‘no’ if not applicable to you)

▪ I did not find it necessary ⃝ yes ⃝ no

▪ I did not think of it ⃝ yes ⃝ no

▪ I thought this professional could not help me ⃝ yes ⃝ no

▪ I was afraid that my employer/supervisor would be informed ⃝ yes ⃝ no

▪ There was no time to discuss this during the appointment ⃝ yes ⃝ no

▪ I found it difficult to discuss ⃝ yes ⃝ no

▪ Other reason, namely: [FILL IN] → go to question 28

26.Has discussing the problems at work during this rheumatology appointment led to actions (such as adjustments to your work, referral to a healthcare or labor professional) in the past 12 months?

□ Yes

□ No → go to question 28

27.What actions have resulted from discussing the problems at work during this rheumatology appointment? (select ‘yes’ if applicable to you and ‘no’ if not applicable to you)

▪ I was referred to another healthcare professional (e.g., physiotherapist, psychologist) ⃝ yes ⃝ no

▪ I was referred to a labor professional (e.g., occupational physician) ⃝ yes ⃝ no

▪ I received advice on how to perform my work with fewer problems ⃝ yes ⃝ no

▪ I received advice to discuss adjustments with my employer/supervisor, such as tasks,

work hours, or work environment (desk, chair, mouse, etc.) ⃝ yes ⃝ no

▪ A workplace visit was conducted ⃝ yes ⃝ no

▪ Other, namely: [FILL IN] ⃝ yes ⃝ no

1. **TREATMENTS AIMED AT YOUR WORK-RELATED ISSUES**

**Healthcare professionals include, among others, the following healthcare providers: general practitioner, physiotherapist, occupational therapist. Occupational healthcare professionals include, among others, the following experts in the field of work-related issues: occupational physician, labour expert.**

28.Have you discussed the problems in performing your work due to your RA/axSpA with a healthcare and/or occupational healthcare professional in the past 12 months?

□ Yes → go to question 30

□ No

29.What were your reasons for NOT discussing your problems in performing your work due to your RA/axSpA with a healthcare and/or labor professional in the past 12 months? (select ‘yes’ if applicable to you and ‘no’ if not applicable to you)

□ I did not find it necessary ⃝ yes ⃝ no

□ I did not think of it ⃝ yes ⃝ no

□ I did not know how or with whom to contact ⃝ yes ⃝ no

□ I thought nobody could help me with this ⃝ yes ⃝ no

□ I was afraid my employer would be informed ⃝ yes ⃝ no

□ I did not have time for this ⃝ yes ⃝ no

□ I did not have money for this ⃝ yes ⃝ no

□ I found it difficult to discuss ⃝ yes ⃝ no

□ Other reason, namely: [FILL IN]

30.With which healthcare and/or occupational healthcare professional(s) have you discussed these work-related problems in the past 12 months? (select ‘yes’ if applicable to you and ‘no’ if not applicable to you)

▪ General practitioner ⃝ yes ⃝ no

▪ Physiotherapist ⃝ yes ⃝ no

▪ Occupational therapist ⃝ yes ⃝ no

▪ Social worker ⃝ yes ⃝ no

▪ Psychologist ⃝ yes ⃝ no

▪ Rehabilitation physician ⃝ yes ⃝ no

▪ Occupational physician ⃝ yes ⃝ no

▪ Occupational physiotherapist ⃝ yes ⃝ no

▪ Occupational health specialist/occupational health physician ⃝ yes ⃝ no

▪ Labour expert/employment specialist ⃝ yes ⃝ no

▪ Insurance physician ⃝ yes ⃝ no

▪ Other, namely: [FILL IN]

Question 31-34 will be asked for each selected professional.

31.How was the appointment with this professional arranged?

□ I specifically made an appointment to discuss the problems in my work due to RA/axSpA

□ I already had appointments scheduled with this professional

□ Other, namely: [FILL IN]

32.Has discussing the problems in your work with this professional led to actions (such as adjustments to your work, referral to a healthcare or labor professional) in the past 12 months?

□ Yes

□ No → go to question 34

33.What actions have resulted from discussing the problems in your work with this professional? (select ‘yes’ if applicable to you and ‘no’ if not applicable to you)

▪ I was referred to another healthcare professional (such as rheumatologist,

physiotherapist) ⃝ yes ⃝ no

▪ I was referred to another labor professional (such as occupational physician,

labour expert) ⃝ yes ⃝ no

▪ I received advice on how to perform my work with fewer problems ⃝ yes ⃝ no

▪ I received a physical training/exercise program to perform my work with fewer

Problems ⃝ yes ⃝ no

▪ I received mental training to perform my work with fewer problems ⃝ yes ⃝ no

▪ I received advice to discuss adjustments with my employer/supervisor, such as tasks,

work hours, or work environment (desk, chair, mouse, etc.) ⃝ yes ⃝ no

▪ A workplace visit was conducted ⃝ yes ⃝ no

▪ Other, namely: [text field]

34.Were the consultations with this professional sufficient for you to discuss and, where possible, alleviate your problems in performing your work?

□ Yes

□ No

□ I would have liked to have more consultations with this professional ⃝ yes ⃝ no

□ I would have also liked to have consultations with other professionals ⃝ yes ⃝ no → if yes, then question 35

35.With which other healthcare and/or occupational healthcare professional(s) would you have preferred to have (also) consultations in the past 12 months to discuss and, where possible, alleviate the problems in performing your work? (select ‘yes’ if applicable to you and ‘no’ if not applicable to you)

▪ General practitioner ⃝ yes ⃝ no

▪ Physiotherapist ⃝ yes ⃝ no

▪ Occupational therapist ⃝ yes ⃝ no

▪ Social worker ⃝ yes ⃝ no

▪ Psychologist ⃝ yes ⃝ no

▪ Rehabilitation physician ⃝ yes ⃝ no

▪ Occupational physician ⃝ yes ⃝ no

▪ Occupational physiotherapist ⃝ yes ⃝ no

▪ Occupational health specialist/occupational health physician ⃝ yes ⃝ no

▪ Labour expert/employment specialist ⃝ yes ⃝ no

▪ Insurance physician ⃝ yes ⃝ no

▪ Other, namely: [FILL IN]

Question 36 will be asked for each selected professional.

36.What were your reasons for NOT visiting this healthcare and/or l occupational healthcare professional in the past 12 months? (select ‘yes’ if applicable to you and ‘no’ if not applicable to you) □ I did not know how or with whom to contact ⃝ yes ⃝ no

□ I was afraid my employer would be informed ⃝ yes ⃝ no

□ I did not have time for this ⃝ yes ⃝ no

□ I did not have money for this ⃝ yes ⃝ no

□ I found it difficult to discuss ⃝ yes ⃝ no

□ Other reason, namely: [FILL IN]

36.Is there anything else you would like to add on this topic? [free text field]

1. **GENERAL HEALTH**

37.EQ-5D-5L (5 items) + VAS thermometer

**G. GENERAL QUESTIONS**

We appreciate it if you would like to answer a few general questions as well. This helps us to describe the group of people who have completed this questionnaire. These data are not reported individually. You can choose to skip these questions if you prefer.

38.What is your year of birth? [Text field]

39.What is your gender?

□ Male

□ Female

□ Non-binary

□ Other, namely:

40.Are you currently participating or have you participated in the Physiotherapy WORKs study?

□ Yes

□ No

41.In what year was your diagnosis of RA or axSpA established by a rheumatologist? [Text field]

42.Do you have any other condition or illness besides RA/axSpA? Please indicate which of the following diseases/conditions you currently have or have had in the past 12 months:

□ Diabetes (also known as diabetes mellitus)

□ Stroke, brain hemorrhage, or cerebral infarction

□ Heart attack

□ Serious heart condition such as heart failure or angina pectoris

□ Form of cancer

□ Migraine or frequent severe headaches

□ Asthma

□ COPD, chronic bronchitis, emphysema

□ Severe or persistent gastrointestinal disorders, lasting more than 3 months

□ Severe or persistent back condition, not related to your rheumatism

□ Severe or persistent condition of the neck or shoulder, not related to your rheumatic condition

□ Allergy

□ High blood pressure

□ Involuntary urine loss, also known as incontinence

□ Cirrhosis of the liver

□ Kidney disease

□ Depression

□ Overweight/obesity

□ Parkinson's disease

□ Multiple sclerosis

□ Other, namely [FILL IN]

43.What is your highest completed education?

□ No or some classes of primary school

□ Primary education

□ Lower vocational education

□ General secondary education

□ Intermediate vocational education

□ Higher general education

□ Higher vocational education

□ Scientific (university) education

You have completed the questionnaire. Thank you for your participation!
