# Supplementary material for: Work-related support for employed and self-employed people with rheumatoid arthritis or axial spondyloarthritis: a cross-sectional online survey of patients
Source: Rheumatol Int. 2024 Jun 20;44(8):1553–65. doi: 10.1007/s00296-024-05643-z (PMC11222234; doi:10.1007/s00296-024-05643-z)
Supplement: Supplementary file 3 — Supplementary file3 (DOCX 43 KB) [file 296_2024_5643_MOESM3_ESM.docx]

**Supplementary table A: Work characteristics and work-related problems of (self-)employed participants with rheumatoid arthritis or axial spondyloarthritis**

|  | **Rheumatoid arthritis, n= 348** | | | **Axial spondyloarthritis, n= 298** | | |
| --- | --- | --- | --- | --- | --- | --- |
|  | *Total,*  *n=348* | *Employed, n=315* | *Self-employed, n=33* | *Total,*  *n=298* | *Employed, n=263* | *Self-employed, n=35* |
| **Employment type; n (%)**  Permanent employment  Temporary employment  Self-employed with personnel  Self-employed without personnel | N= 348  284 (81.6%)  31 (8.9%)  4 (1.1%)  29 (8.3%) | N= 315  284 (90.2%)  31 (9.8%)  -  - | N= 33  -  -  4 (12.1%)  29 (87.9%) | N= 298  235 (78.9%)  28 (9.4%)  4 (1.3%)  31 (10.4%) | N= 263  235 (89.4%)  28 (10.6%)  -  - | N= 35  -  -  4 (11.4%)  31 (88.6%) |
| **Top-3 job sector; n (%)**  Healthcare  Education and training  Retail and sales | N= 348  120 (34.5%)  42 (12.1%)  37 (10.6%) | N= 315  113 (35.9%)  39 (12.4%)  36 (11.4%) | N= 33  7 (21.2%)  3 (9.1%)  1 (3.0%) | N= 298  121 (40.6%)  34 (11.4%)  27 (9.1%) | N= 263  106 (40.3%)  32 (12.2%)  23 (8.7%) | N= 35  15 (42.6%)  2 (5.7%)  4 (11.4%) |
| **Occupational class; n (%)**  White-collar  Blue-collar | N=339  316 (93.2%)  23 (6.8%) | N= 306  284 (92.8%)  22 (7.2%) | N= 33  32 (97.0%)  1 (3.0%) | N= 288  262 (91.0%)  26 (9.0%) | N= 255  232 (91.0%)  23 (9.0%) | N= 33  30 (90.9%)  3 (9.1%) |
| **Size of company; n (%)**  <10 employees  10 to 50 employees  51 to 250 employees  >250 employees | N= 348  51 (14.7%)  71 (20.4%)  53 (15.2%)  173 (49.7%) | N= 315  24 (7.6%)  69 (21.9%)  52 (16.5%)  170 (54.0%) | N= 33  27 (81.8%)  2 (6.1%)  1 (3.0%)  3 (9.1%) | N= 298  56 (18.8%)  40 (13.4%)  53 (17.8%)  149 (50.0%) | N= 263  25 (9.5%)  38 (14.4%)  52 (19.8%)  148 (56.3%) | N= 35  31 (88.6%)  2 (5.7%)  1 (2.9%)  1 (2.9%) |
| **Working hours; n (%)**  <12 hours/week  12 to 24 hours/week  25 to 36 hours/week  >36 hours/week | N= 348  18 (5.2%)  131 (37.6%)  130 (37.4%)  69 (19.8%) | N= 315  17 (5.5%)  115 (36.5%)  123 (39.0%)  60 (19.0%) | N= 33  1 (3.0%)  16 (48.5%)  7 (21.2%)  9 (27.3%) | N= 298  19 (6.4%)  113 (37.9%)  107 (35.9%)  59 (19.8%) | N= 263  14 (5.3%)  106 (40.3%)  94 (35.7%)  49 (18.6%) | N= 35  5 (14.3%)  7 (20.0%)  13 (37.1%)  10 (28.6%) |
| **Work-related problems in the past 12 months due to the disease; n (%)**  Yes | N= 329  313 (95.1%) | N= 296  281 (94.9%) | N= 33  32 (97.0%) | N= 288  276 (95.8%) | N= 256  245 (95.7%) | N= 32  31 (96.9%) |
| **Type of work-related problems due to disease; n (%)**  *(Multiple answers possible)*  Fatigue  Pain in joints/muscles  Stiffness of joints/muscles  Morning or starting stiffness  Difficulty moving  Swollen joints | N= 329  299 (95.5%)  293 (93.6%)  283 (90.4%)  251 (80.2%)  246 (78.6%)  172 (55.0%) | N= 296  268 (95.4%)  261 (92.9%)  255 (90.7%)  225 (80.1%)  219 (77.9%)  155 (55.2%) | N= 33  31 (93.9%)  32 (100.0%)  28 (87.5%)  26 (81.3%)  27 (84.8%)  17 (53.1%) | N= 288  264 (95.7%)  259 (93.8%)  242 (87.7%)  242 (87.7%)  219 (79.3%)  73 (26.4%) | N= 256  235 (95.9%)  230 (93.9%)  215 (87.8%)  212 (86.5%)  196 (80.0%)  67 (27.3%) | N= 32  29 (93.5%)  29 (93.5%)  27 (87.1%)  30 (96.8%)  23 (74.2%)  6 (17.1%) |
| **Currently on sick leave due to disease; n (%)**  Yes, on partial sick leave  Yes, on full sick leave | N=313  31 (9.9%)  20 (6.4%) | N= 281  29 (10.3%)  20 (7.2%) | N= 32  2 (6.2%)  - | N= 276  27 (10.9%)  23 (9.2%) | N= 245  25 (10.2%)  23 (9.4%) | N= 31  2 (6.5%)  - |
| **Duration of current sick leave due to disease; n (%)**  <6 months  6 to 12 months  >12 months | N=51  17 (33.3%)  18 (35.3%)  16 (31.4%) | N=49  17 (34.7%)  16 (32.7%)  16 (32.7%) | N=2  -  2  - | N= 50  22 (44.0%)  14 (28.0%)  14 (28.0%) | N= 48  22 (45.8%)  13 (27.1%)  13 (27.1%) | N=2  -  1  1 |
| **Work Ability Index-Single Item Scale (WAS),**  **range 0 to 10; Mean (SD)**  **WAS categories; n (%)**  Poor work ability (0-5)  Moderate work ability (6-7)  Good work ability (8-9)  Excellent work ability (10) | N= 329  6.4 (1.8)  100 (30.4%)  160 (48.6%)  66 (20.1%)  3 (0.9%) | N= 296  6.5 (1.8)  90 (30.4%)  139 (47.0%)  64 (21.6%)  3 (1.0%) | N= 33  5.9 (1.4)  10 (30.3%)  21 (63.6%)  2 (6.1%)  - | N= 288  6.3 (1.9)  96 (33.3%)  138 (47.9%)  52 (18.1%)  2 (0.7%) | N= 256  6.4 (1.9)  84 (32.8%)  123 (48.0%)  48 (18.8%)  1 (0.4%) | N= 32  5.8 (1.9)  12 (37.5%)  15 (46.9%)  4 (12.5%)  1 (3.1%) |

**Supplementary table B: Experiences with discussing work-related problems with the rheumatologist or specialized rheumatology nurse**

|  | **Participants with rheumatoid arthritis and work-related problems, n= 313** | | **Participants with axial spondyloarthritis and work-related problems, n= 276** | |
| --- | --- | --- | --- | --- |
|  | *Employed, n=281* | *Self-employed, n=32* | *Employed, n=245* | *Self-employed, n=31* |
| **Rheumatology consultation in the last 12 months; n (%)**  Yes, with rheumatologist  Yes, with specialized rheumatology nurse | N= 270  209 (77.4%)  53 (20.0%) | N= 32  26 (81.3%)  5 (15.6%) | N= 234  185 (79.1%)  30 (12.8%) | N= 30  24 (80.0%)  2 (6.7%) |
| **Discussed work-related problems with the rheumatologist or specialized rheumatology nurse; n (%)**  Yes | N= 262  152 (58.0%) | N= 31  16 (51.6%) | N= 215  126 (58.6%) | N= 26  15 (57.7%) |
| **Reasons for not discussing work-related problems with the rheumatologist or specialized rheumatology nurse; n (%)**  *(Multiple answers possible)*  I did not think this was necessary  I had not thought of this  I did not think they could help me with this  I found it difficult to discuss this  I did not had time for this  I was afraid that my employer would be informed | N= 110  73 (66.4%)  57 (51.8%)  39 (35.5%)  28 (25.5%)  26 (23.6%)  6 (5.5%) | N= 15  9 (60.0%)  8 (53.9%)  5 (33.3%)  4 (26.7%)  2 (13.3%)  - | N= 89  55 (61.8%)  43 (48.3%)  32 (36.0%)  19 (21.3%)  16 (18.0%)  6 (6.7%) | N= 11  6 (54.5%)  6 (54.5%)  7 (63.6%)  2 (18.2%)  7 (63.6%)  1 (9.1%) |
| **Advices or actions arisen from discussing work-related problems with the rheumatologist or specialized rheumatology nurse; n (%)**  Yes | N= 152  38 (25.0%) | N= 16  2 (12.5%) | N= 126  44 (34.9%) | N= 15  6 (40.0%) |
| **Advices or actions resulting from discussions with the rheumatologist or specialized rheumatology nurse; n (%)**  *(Multiple answers possible)*  I have been advised to discuss adjustments in working tasks/hours/environment with my employer  I have been referred to a different HCP  I received advice on how to perform my job with fewer problems  I have been referred to an occupational HCP  A workplace examination has been carried out | N= 38  20 (52.6%)  19 (50.0%)  19 (50.0%)  12 (31.6%)  6 (15.8%) | N= 2  -  -  1 (50.0%)  -  - | N= 44  26 (59.1%)  24 (54.5%)  17 (38.6%)  16 (36.4%)  5 (11.4%) | N= 6  1 (16.7%)  2 (33.3%)  2 (33.3%)  1 (16.7%)  1 (16.7%) |

*HCP: healthcare professional*

**Supplementary table C: Experiences with discussing work-related problems with other HCPs**

|  | **Participants with rheumatoid arthritis and work-related problems, n= 313** | | **Participants with axial spondyloarthritis and work-related problems, n= 276** | |
| --- | --- | --- | --- | --- |
|  | *Employed, n=281* | *Self-employed, n=32* | *Employed, n=245* | *Self-employed, n=31* |
| **Discussed work-related problems with another (occupational) HCP; n (%)**  Yes | N= 247  98 (39.7%) | N= 30  4 (13.3%) | N= 211  106 (50.2%) | N= 26  11 (42.3%) |
| **Which (occupational) HCP is consulted for work-related problems; n (%)**  *(Multiple answers possible)*  HCPs:  Physiotherapist  General practitioner  Occupational therapist  Psychologist  Social worker  Physiatrist  Occupational HCPs:  Occupational physician  Labour expert  Specialized occupational physiotherapist  Insurance physician | N= 98  66 (67.3%)  36 (36.7%)  19 (19.4%)  22 (22.4%)  14 (14.3%)  11 (11.2%)  59 (60.2%)  20 (20.4%)  10 (10.2%)  6 (6.1%) | N= 4  3 (75.0%)  1 (25.0%)  1 (25.0%)  1 (25.0%)  -  -  -  -  -  - | N= 106  75 (70.8%)  52 (49.1%)  13 (12.3%)  29 (27.4%)  12 (11.3%)  6 (5.7%)  55 (51.9%)  16 (15.1%)  6 (5.7%)  2 (1.9%) | N= 11  10 (90.9%)  1 (9.1%)  -  3 (27.3%)  1 (9.1%)  -  -  1 (9.1%)  -  1 (9.1%) |
| **Reasons for not discussing work-related problems with (occupational) HCP; n (%)**  *(Multiple answers possible)*  I did not think this was necessary  I had not thought of this  I found it difficult to discuss this  I did not think anybody could help me with this  I did not know who to consult  I did not had time for this  I was afraid that my employer would be informed  I did not had money for this | N= 149  98 (65.8%)  59 (39.6%)  54 (36.2%)  47 (31.5%)  43 (28.9%)  28 (18.8%)  26 (17.4%)  20 (13.4%) | N= 26  12 (46.2%)  11 (42.3%)  4 (15.4%)  9 (34.6%)  7 (26.9%)  3 (11.5%)  1 (3.8%)  2 (7.7%) | N= 105  56 (53.3%)  34 (32.4%)  45 (42.9%)  41 (39.0%)  38 (36.2%)  18 (17.1%)  19 (18.1%)  14 (13.3%) | N= 15  11 (73.3%)  6 (40.0%)  4 (26.7%)  8 (53.3%)  7 (46.7%)  7 (46.7%)  -  3 (20.0%) |
| **Advices or actions arisen from discussing work-related problems with the (occupational) HCP; n (%)**  Yes | N= 98  49 (50.0%) | N= 4  2 (50.0%) | N= 106  60 (56.6%) | N= 11  6 (54.6%) |
| **Advices or actions resulting from discussions with the HCP; n (%)**  *(Multiple answers possible)*  I have been advised to discuss adjustments in working tasks/hours/environment with my employer  I received advice on how to perform my job with fewer problems  I have been referred to a (different) HCP  I received a physical exercise program to be able to perform my job with fewer problems  I have been referred to an (different) occupational HCP  I received mental training to be able to perform my job with fewer problems  A workplace examination has been carried out | N= 49  35 (71.4%)  33 (67.3%)  18 (36.7%)  17 (34.7%)  15 (30.6%)  12 (24.5%)  12 (24.5%) | N= 2  -  2 (100.0%)  1 (50.0%)  2 (100.0%)  -  -  - | N= 60  36 (60.0%)  34 (56.6%)  27 (45.0%)  17 (28.3%)  16 (26.6%)  14 (23.3%)  6 (10.0%) | N= 6  1 (16.6%)  5 (83.3%)  -  5 (83.3%)  -  1 (16.6%)  - |
| **Was discussing the work-related problems with the (occupational) HCP sufficient to reduce the work-related problems; n (%)**  Yes | N= 98  63 (64.3%) | N= 4  3 (75.0%) | N= 106  72 (67.9%) | N= 11  6 (54.6%) |

*HCP: healthcare professional*

**Supplementary table D: Experiences of employed participants with work-related problems with discussing work-related problems with the employer***

|  | **Employees with rheumatoid arthritis and work-related problems, n= 281** | **Employees with axial spondyloarthritis and work-related problems, n= 245** |
| --- | --- | --- |
| **Discussed work-related problems with the employer; n (%)**  Yes | N= 277  189 (68.2%) | N= 237  163 (68.8%) |
| **Reasons for not discussing work-related problems with the employer; n (%)**  *(Multiple answers possible)*  I didn’t think this was necessary  I found it difficult to discuss this  I was afraid of possible adverse consequences for my job or employment contract  I thought my employer couldn’t help me with this  I hadn’t thought of this | N= 88  63 (71.6%)  48 (54.5%)  43 (48.9%)  37 (42.0%)  22 (25.0%) | N= 74  45 (60.8%)  43 (58.1%)  44 (59.5%)  29 (39.2%)  12 (16.2%) |
| **Advices or actions arisen from discussing work-related problems with the employer; n (%)**  Yes | N= 189  105 (55.6%) | N= 163  93 (57.1%) |
| **Advices or actions resulting from discussions with the employer; n (%)**  *(Multiple answers possible)*  I received advice to consult an occupational HCP  I received advice to consult a HCP  I perform fewer tasks in a working day  I perform different tasks  I work less hours: by reducing the number of hours in my employment contract  I work less hours: by reducing taking vacation hours  I work less hours: other  I work at different times  My work environment is adjusted  I have found another job or am currently looking for another job | N= 105  55 (52.4%)  51 (48.6%)  45 (42.9%)  43 (41.0%)  17 (37.0%)  5 (10.9%)  21 (45.7%)  37 (35.2%)  36 (34.3%)  14 (13.3%) | N= 93  45 (48.4%)  42 (45.2%)  37 (39.8%)  27 (29.0%)  15 (42.9%)  5 (14.3%)  18 (51.4%)  31 (33.3%)  42 (45.2%)  7 (7.5%) |

**Not applicable for self-employed participants HCP: healthcare professional*
